# Evaluating the Accuracy of Two Intraoral Scanning Systems in Reproducing Implant Positions for Prosthetic Fabrication: A Cross-Sectional Comparative Study

**DOI:** 10.7759/cureus.98548

**Published:** 2025-12-05

**Authors:** Shankar S Menon, Biju Balakrishnan, Arun Kurumathur Vasudevan

**Affiliations:** 1 Periodontics, Amrita School of Dentistry, Amrita Vishwa Vidyapeetham, Kochi, IND

**Keywords:** accuracy comparison, dentsply sirona, implant positions, intraoral scanning systems, medit i700, prosthetic fabrication

## Abstract

Background: The integration of digital technologies in implant dentistry has enhanced the precision of prosthetic fabrications by minimizing errors associated with traditional impression methods. However, variations in the accuracy of intraoral scanning systems can impact the fit and function of implant-supported prostheses. This study compared the accuracy of the Medit i700 (Medit Corp, Seoul, Korea) and Dentsply Sirona intraoral scanning systems in replicating implant positions, using direct intraoral measurements as the reference standard.

Methods: Six patients with 10 implant sites were included. Inclusion criteria involved stable implants with adjacent natural teeth. Direct intraoral distances from scan bodies to adjacent teeth were measured using a digital caliper. Digital scans were obtained for every site with both the Medit i700 and Dentsply Sirona (Dentsply Sirona, Bensheim, Germany) intraoral scanning systems, and three-dimensional (3D)-printed models were fabricated for measurement. To assess the accuracy of reproducibility in models, intercuspal distances and distances between the scan bodies and the nearest portion of adjacent teeth were measured. Accuracy was evaluated using intraclass correlation coefficient (ICC) and Bland-Altman analysis.

Results: For distances from scan bodies to adjacent teeth, the mean intraoral distance was 8.62 ± 4.28 mm. Medit i700 measurements (8.60 ± 4.25 mm) showed ICC = 1.00 and bias = 0.02 mm (limits: -0.10 to 0.14 mm). Dentsply Sirona (8.56 ± 4.34 mm) had ICC = 0.999 and bias = 0.05 mm (limits: -0.32 to 0.43 mm), indicating greater variability. For intercuspal distances, measurements were similar (Medit: 5.95 ± 0.77 mm; Sirona: 5.93 ± 0.76 mm; ICC = 0.999).

Conclusion: The Medit i700 demonstrated superior accuracy over the Dentsply Sirona system, due to its advanced features and recent manufacturing. This supports its preference for precise implant prosthetics.

## Introduction

The field of implant dentistry has undergone a significant transformation with the integration of digital technologies in impression taking for prosthetic restorations. Traditional methods involving elastomeric materials have long been the standard, but they are prone to distortions from material shrinkage, improper handling, or patient movement [1]. Intraoral scanning (IOS) systems have emerged as a reliable alternative, capturing three-dimensional (3D) images of the oral cavity through optical principles such as confocal microscopy or triangulation [2]. The evolution of IOS systems dates back to the 1980s with the introduction of the CEREC system (Dentsply Sirona, Bensheim, Germany), which utilized structured light for image acquisition [3]. Subsequent advancements have included the shift to powder-free scanning, color imaging, wireless designs, and integration of artificial intelligence (AI) for artifact removal, thereby enhancing usability, patient acceptance, and overall efficiency [2,4].

In implant dentistry, accurate reproduction of implant positions is paramount for fabricating prostheses that ensure passive fit, optimal occlusion, and longevity. Misalignments as small as 50-100 micrometers (μm) can induce stress on the implant-bone interface, leading to biological or mechanical failures [5]. Intraoral scanning systems facilitate direct digitization of implant scan bodies, eliminating intermediate steps like gypsum casts that introduce errors [6]. Studies have shown that digital impressions can achieve trueness (closeness to true dimensions) and precision (repeatability) comparable to or better than conventional techniques for single implants or short spans [7]. For instance, recent evaluations of new-generation IOS systems report trueness values below 50 μm for quadrant scans [8]. A 2025 umbrella review highlighted that contemporary IOS models, such as TRIOS 3 (3Shape A/S, Copenhagen, Denmark) and Primescan (Dentsply Sirona, Bensheim, Germany), exhibit high accuracy for single-unit and short-span implant restorations in dentate arches, although limitations persist in edentulous cases [9].

Several factors influence the accuracy of IOS systems, including scanner type, scanning strategy, implant scan body design, inter-implant distance, arch span, and implant angulation [10]. The literature highlights variations among devices; for example, the TRIOS series often exhibits high trueness in full-arch scans, while others like the CS 3600 (Carestream Dental, Atlanta, Georgia, USA) perform well in precision but may falter in angulated implants [11]. Comparative in vitro studies have assessed multiple IOS systems for implant impressions. One investigation found the Primescan (Dentsply Sirona, Bensheim, Germany) to have superior trueness (35.75 ± 26.08 μm) over the Medit i700 (Medit Corp, Seoul, Korea) in edentulous models with four implants [12]. Conversely, another study reported the Medit i700 as having the least deviation (62 ± 16 μm) in All-on-Four models with 30° angulations, outperforming the Primescan [13]. These discrepancies may stem from model complexity, angulation effects, or software versions. Recent research from 2025 further emphasizes that implant placement level and scan body height significantly affect digital impression accuracy, with tissue-level implants and shorter scan bodies yielding better trueness, and the Medit i700 outperforming the Primescan in certain configurations [14]. Additionally, implant angulation has been shown to negatively impact trueness in IOS without auxiliary devices, with deviations increasing in distally tilted setups [15].

The Medit i700, released in 2021, incorporates high-speed cameras, AI-driven artifact removal, and a depth of field up to 20 mm, claiming full-arch accuracy of 11 μm [13]. In contrast, the Dentsply Sirona scanner (Primescan or Omnicam models from 2019) uses dynamic deep scanning technology, processing over 1 million 3D points per second [10]. However, older models may lack the latest algorithmic enhancements, potentially affecting performance in challenging scenarios like posterior regions or subgingival margins [7]. Cost differences also play a role; premium devices like the Medit i700 often justify higher prices through superior hardware, which could translate to better clinical outcomes [13]. A 2025 in vitro study on All-on-4 implants found that while conventional impressions offer superior trueness, IOS like TRIOS 5 and Runyes 3DS (Runyes Medical Instrument Co., Ltd., Ningbo, China) provide higher precision, although deviations may exceed clinical thresholds without additional strategies [16].

Despite abundant research on the accuracy of IOS systems, few studies directly compare devices with differing manufacturing dates and costs in the context of single-implant prosthetics. A systematic review emphasized that implant scan body position and scanner generation significantly impact full-arch accuracy, with newer systems showing reduced deviations [5]. Gaps persist in evaluating how these factors affect measurements like scan body-to-tooth distances, which are crucial for prosthesis design. Moreover, while in vitro studies dominate, they often overlook clinical variables such as saliva or soft-tissue mobility [6]. Emerging evidence from 2025 reviews underscores the multifactorial nature of IOS accuracy, including operator experience, scan body material, and environmental factors, advocating for optimized workflows in digital implant prosthodontics [17].

This cross-sectional comparative study addresses these gaps by comparing the Medit i700 and Dentsply Sirona IOS systems in reproducing implant positions for prosthetic fabrication. By using direct intraoral measurements as the gold standard, we assessed whether the more advanced and costlier Medit i700 provides superior accuracy over the older Dentsply Sirona model. Findings could guide clinicians in selecting IOS systems for efficient implant workflows, ultimately improving patient outcomes in restorative dentistry.

## Materials and methods

This cross-sectional comparative study involved patients undergoing implant prosthetic treatment in the Department of Periodontics, Amrita School of Dentistry, Kochi, Kerala. Ethical approval was obtained from the Institutional Review Board (IRB), Amrita School of Medicine, Amrita Vishwa Vidyapeetham, Kochi, India (approval no. ECASM-AIMS-2025-359, dated: 13-10-2025), and all participants provided informed consent.

Patients were selected based on the following inclusion criteria: adults aged 18-70 years with osseointegrated MIS implants (MIS Implants Technologies Ltd.) in stable condition, healthy peri-implant tissues (probing depth <4 mm, no bleeding on probing), adjacent natural teeth for reference, and good oral hygiene (plaque index <20%). Exclusion criteria included implant mobility, severe periodontitis, malocclusion affecting measurements, pregnancy, or uncontrolled systemic diseases like diabetes. The objective of the study is primarily to compare the two IOS systems which differ in manufacturer type and date, generation, etc.

This was a clinical comparative study. Sample size was calculated a priori using G*Power 3.1 software (Heinrich-Heine-Universität Düsseldorf, Düsseldorf, Germany), with the primary outcome being the absolute difference in scan body-to-adjacent tooth distance between the gold-standard intraoral measurement and the 3D-printed model measurement. Thereupon, a clinically acceptable difference of ≤0.10 mm with a standard deviation of 0.09 mm was assumed. Using a two-tailed paired t-test design, α = 0.05, power = 80%, and anticipating a moderate-to-large effect size (Cohen’s d ≈ 0.9), the minimum required sample size was eight implant sites. To account for possible measurement errors or dropouts and to increase the robustness of Bland-Altman analysis, we included 10 implant sites from six patients. This sample size also allowed reliable estimation of the intraclass correlation coefficient (ICC) with a 95% confidence interval width of approximately ±0.15 when the expected ICC is close to 1.0.

A total of 10 implant sites were thus evaluated across six patients. For each site, a titanium scan body was attached to the MIS implant at 20 Newton-centimeters (Ncm) torque using a calibrated torque wrench. Direct intraoral measurements (gold standard) were performed by a single calibrated operator using a digital vernier caliper (Mitutoyo (Mitutoyo Corporation, Kawasaki, Japan), accuracy ±0.01 mm). The distance between the scan body apex and the adjacent tooth's mesial or distal surface was measured.

**Figure 1:**
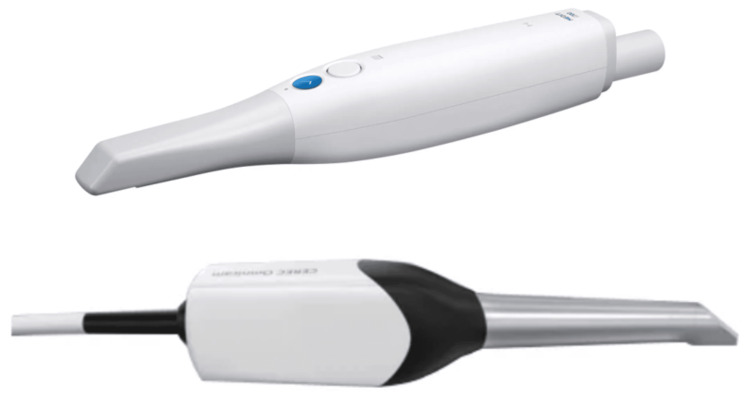
Medit i700 scanner (above) and Dentsply Sirona CEREC Omnicam scanner (below) Figure credits: Dr. Shankar S Menon.

Digital impressions were then acquired using two IOS systems: Medit i700 (Medit Corp, Seoul, Korea; manufactured April 2021) and Dentsply Sirona CEREC Omnicam (Dentsply Sirona, Bensheim, Germany; manufactured 2019) (as shown in Figure 1). Scans were conducted in a controlled environment following manufacturer guidelines. Each scanner was calibrated prior to use, and scans were repeated if artifacts were detected visually.

**Figure 2:**
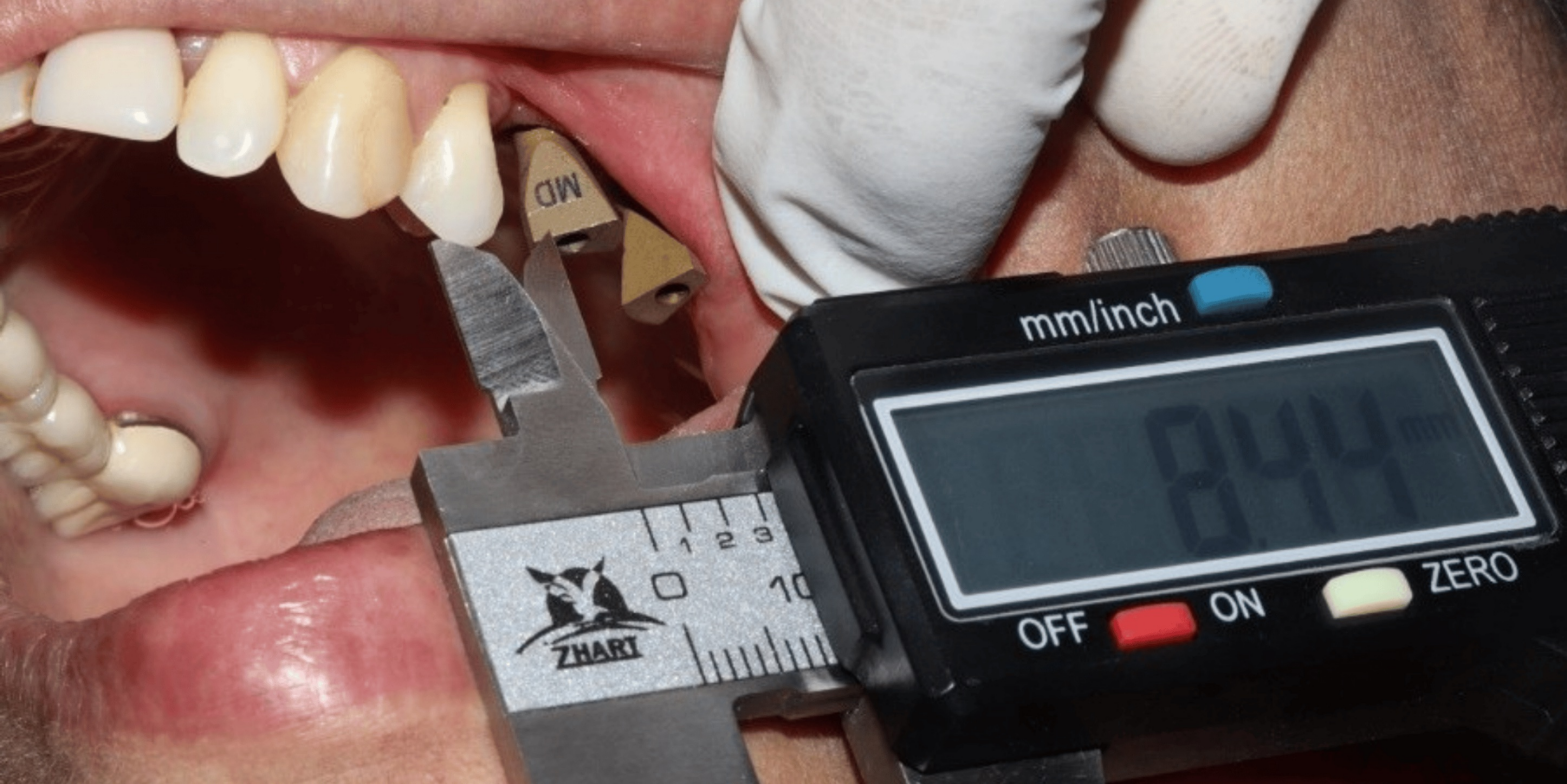
The process of measuring the distance between the nearest part of the tooth surface to the scan body using a calibrated digital caliper intraorally Figure credits: Dr. Shankar S Menon.

The Standard Tessellation Language (STL) files from both scanners were exported and imported into computer-aided design (CAD) software (Exocad DentalCAD; Exocad GmbH, Darmstadt, Germany) for alignment and processing. Three-dimensional models were printed using a stereolithography printer. On these printed models, the distance between the scan body and the adjacent tooth was remeasured using the same caliper protocol (as shown in Figure 2).

**Figure 3:**
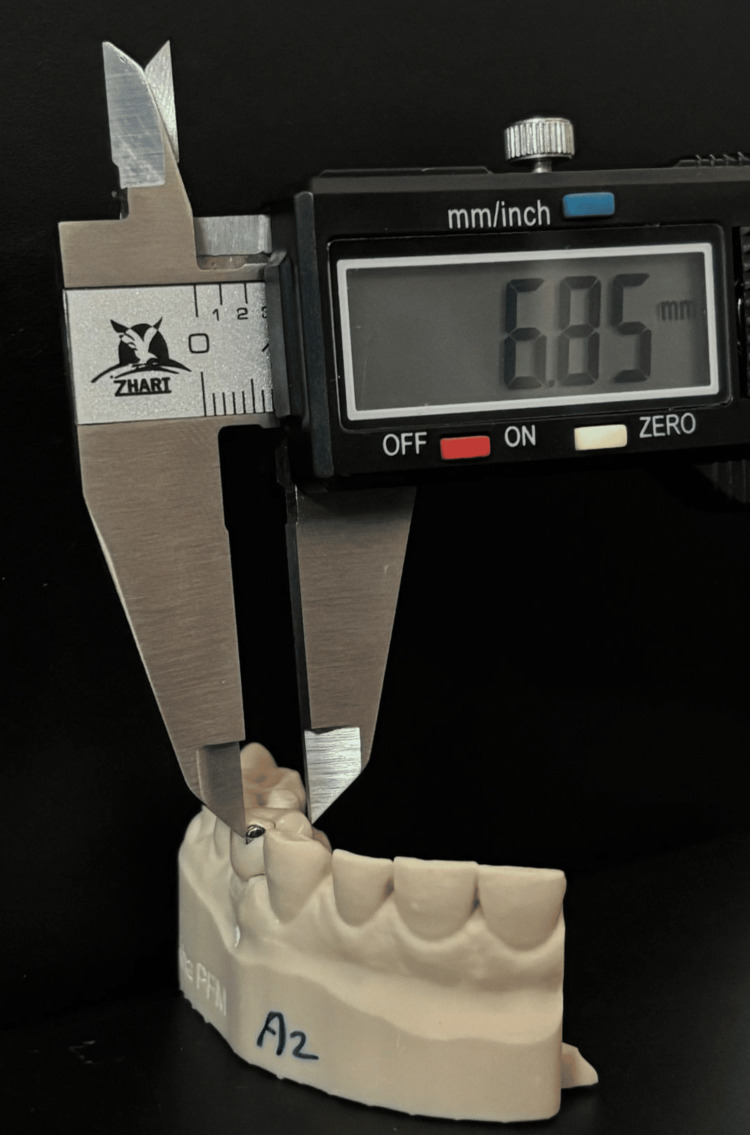
The process of measuring the intercuspal distance using a calibrated digital caliper on 3D printed model Figure credits: Dr. Shankar S Menon.

Additionally, the intercuspal distance between cusps of adjacent teeth was measured on the models from both scanners to assess inter-scanner consistency (as shown in Figure 3).

Statistical analysis

All measurements were blinded to the scanner type. Data were analyzed using IBM SPSS version 20.0 (SPSS Inc., Chicago, USA). Normality was confirmed via Shapiro-Wilk tests. Descriptive statistics included means ± standard deviations. Accuracy was assessed using ICC between intraoral (gold standard) and scanner-derived measurements for scan body-to-tooth distances. Bland-Altman plots evaluated bias and limits of agreement (LoA). For intercuspal distances, paired t-tests compared inter-scanner differences.

## Results

The study included measurements from 10 implant sites across six patients, with no adverse events or data exclusions. Baseline characteristics of the study sample have been described in Table 1.

**Table 1:** Baseline characteristics of the study participants Table credits: Dr. Shankar S Menon. SD: standard deviation.

Characteristic	Value
Age (years), mean ± SD (range)	46.3 ± 12.8 (28-65)
Gender, n (%)	
Male	4 (66.7%)
Female	2 (33.3%)
Implant location, n	
Maxilla	7 sites
Mandible	3 sites
Implant region, n	
Premolar	4
Molar	6
Time since implant placement (months), mean ± SD	8.4 ± 2.1 (6-13)
Implant depth, n	
Bone-level	10 (100%)
Peri-implant probing depth (mm), mean ± SD	2.6 ± 0.6
Bleeding on probing, n (%)	0 (0%)
Plaque index (Silness-Löe), mean ± SD	0.3 ± 0.4

Descriptive statistics for scan body-to-tooth distances and intercuspal distances are presented in Table 2.

**Table 2:** Measurements obtained across implant sites (distance between scan body and adjacent part (mesial/distal) of tooth; and intercuspal distance) Table credits: Dr. Shankar S Menon.

		Distance between scan body and adjacent part of tooth			Intercuspal distance	
Sl. No.	Region	Intraorally	On 3D printed model		On 3D printed model	
			Medit i700	Dentsply Sirona	Medit i700	Dentsply Sirona
1	"25"	8.44 mm	8.43 mm	8.39 mm	5.75 mm	5.75 mm
	"26"	18.55 mm	18.50 mm	18.66 mm	6.54 mm	6.53 mm
	"36"	10.90 mm	10.92 mm	11.11 mm	6.42 mm	6.40 mm
2	"26"	5.09 mm	5.11 mm	5.28 mm	6.15 mm	6.16 mm
	"27"	12.22 mm	12.04 mm	11.96 mm	6.25 mm	6.25 mm
3	"45"	5.34 mm	5.39 mm	5.04 mm	5.17 mm	5.15 mm
	"47"	5.37 mm	5.38 mm	5.39 mm	6.85 mm	6.85 mm
4	"24"	5.92 mm	5.90 mm	5.62 mm	4.89 mm	4.88 mm
5	"16"	8.34 mm	8.30 mm	8.30 mm	6.71 mm	6.60 mm
6	"14"	5.98 mm	5.98 mm	5.87 mm	4.74 mm	4.74 mm

**Figure 4:**
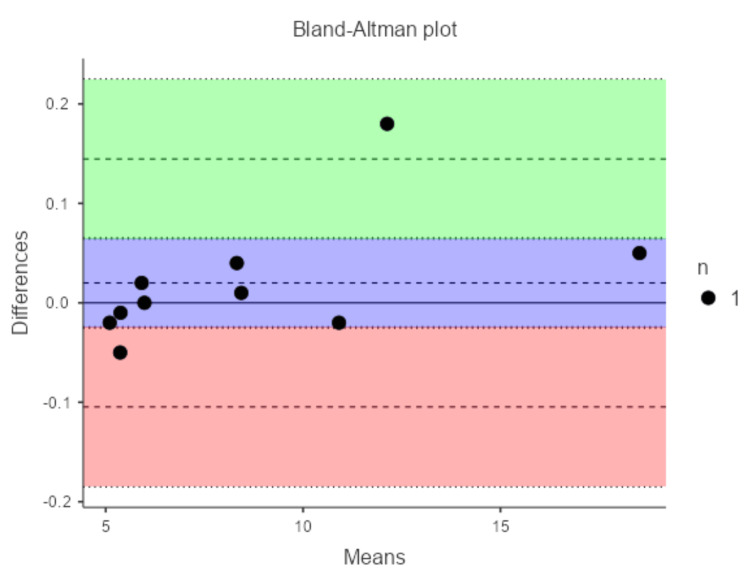
Graph showing Bland-Altman analysis for Medit i700 scanner Figure credits: Dr. Shankar S Menon.

The mean intraoral measurement for scan body-to-tooth distance was 8.62 ± 4.28 mm (range: 5.09-18.55 mm). Medit i700 model measurements averaged 8.60 ± 4.25 mm (range: 5.11-18.50 mm), while Dentsply Sirona averaged 8.56 ± 4.34 mm (range: 5.04-18.66 mm). Differences from the gold standard were minimal for both scanners but smaller for Medit i700 (mean difference: -0.02 ± 0.06 mm, range: -0.18 to 0.05 mm) compared to Dentsply Sirona (-0.05 ± 0.19 mm, range: -0.30 to 0.21 mm). The ICC for Medit i700 versus intraoral was 1.00, indicating excellent agreement. For Dentsply Sirona, ICC was 0.999, also excellent but slightly lower. Bland-Altman analysis for Medit i700 showed a bias of 0.02 mm, with LoA from -0.10 mm to 0.14 mm, as shown in Figure 4.

**Figure 5:**
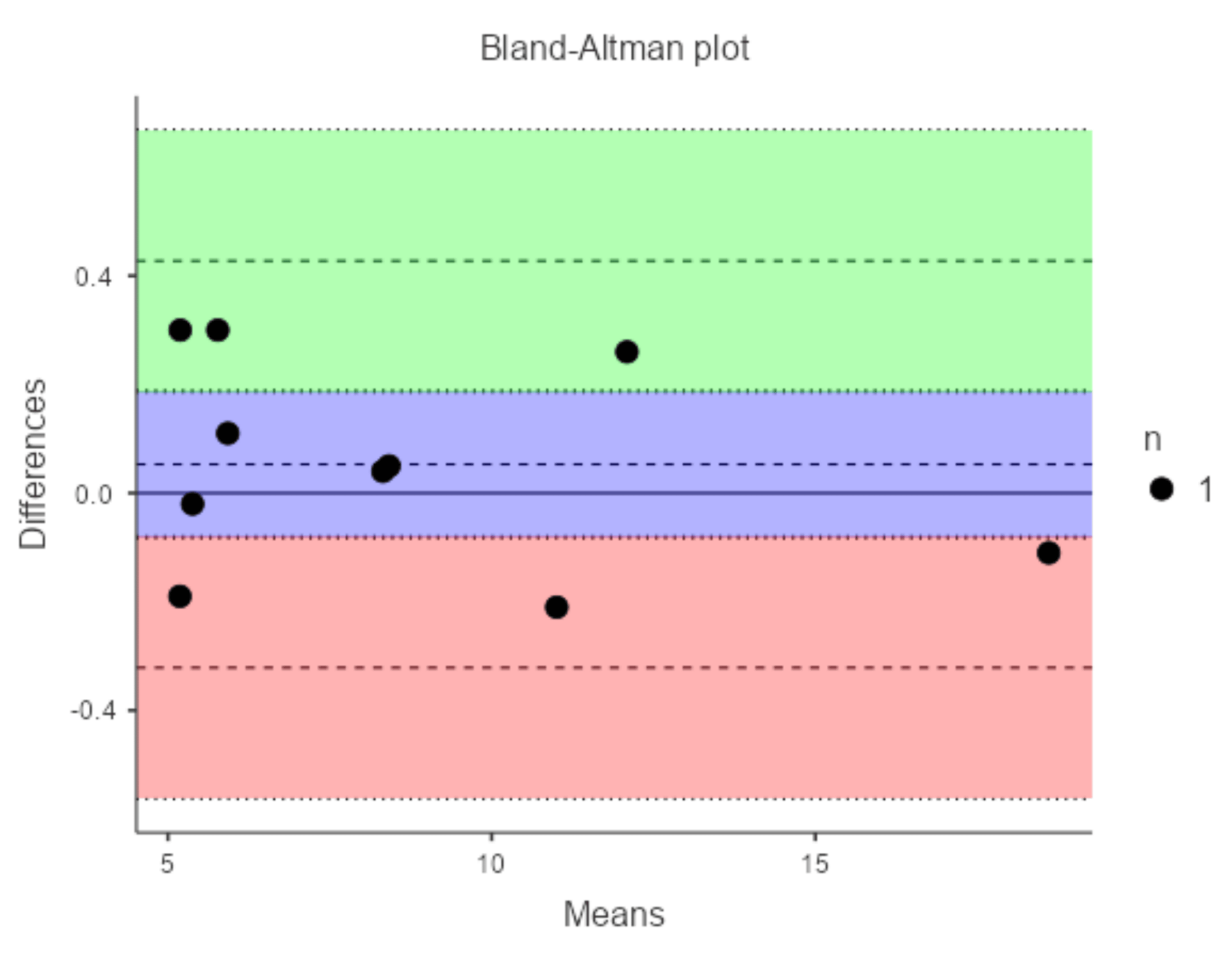
Graph showing Bland-Altman analysis for Dentsply Sirona CEREC Omnicam scanner Figure credits: Dr. Shankar S Menon.

For Dentsply Sirona, bias was 0.05 mm, with wider LoA: lower -0.32 mm and upper 0.43 mm. These wider limits suggest greater variability in the Dentsply Sirona scanner. The analysis is shown in Figure 5.

For intercuspal distances on models, means were 5.95 ± 0.77 mm for Medit i700 (range: 4.74-6.85 mm) and 5.93 ± 0.76 mm for Dentsply Sirona (range: 4.74-6.85 mm). The mean inter-scanner difference was 0.02 ± 0.03 mm (range: -0.01 to 0.11 mm), with no significant difference (p=0.12). ICC between scanners was 0.999, indicating near-perfect concordance.

## Discussion

The outcomes of this study reveal that the intraoral scanning system from a newer generation, exemplified by Medit i700 (manufactured in April 2021), exhibits enhanced accuracy in replicating implant positions relative to an older generation model, such as the Dentsply Sirona scanner (manufactured in 2019). This is evidenced by superior intraclass correlation coefficient values and narrower limits of agreement when compared against intraoral gold standard measurements. For the distance between the scan body and the adjacent part of the tooth, the newer system displayed a negligible bias of 0.02 mm with limits spanning -0.10 to 0.14 mm, in contrast to the older system's bias of 0.05 mm and broader limits from -0.32 to 0.43 mm. These results imply that newer scanning technologies may offer greater dependability in documenting spatial configurations essential for implant-based prosthetic construction, potentially reducing the need for adjustments during prosthesis placement and lowering the risk of complications from ill-fitting restorations [5,13]. The superior performance of the Medit i700 aligns with its advanced features, such as high-speed cameras and AI-driven processing, which minimize distortions in clinical settings [2,14].

These observations support the hypothesis that advancements in successive generations of IOS systems contribute to improved performance. Newer models typically integrate refinements in optical quality, such as enhanced resolution sensors and expanded depth of field, alongside AI algorithms that facilitate automated artifact elimination and efficient data processing [2]. Furthermore, these systems demonstrate superior capability in managing complex anatomical features, including undercuts and tilted scan bodies or implants, through optimized scanning paths and adaptive light projection techniques that minimize distortions in challenging areas [8]. The disparity in manufacturing dates highlights how rapid technological evolution in digital dentistry can render earlier devices less optimal for precision-demanding applications [10,15]. For instance, the Medit i700's lightweight design (245g) and reversible tip enhance maneuverability, particularly in posterior regions, compared to the bulkier Dentsply Sirona Primescan (around 457g), which may contribute to reduced operator fatigue and improved scan consistency [13]. Additionally, faster scanning speeds of the Medit i700 (up to 70 frames per second, full-arch in 18-40 seconds) versus the Primescan (under 60 seconds) streamline workflows, potentially decreasing patient discomfort and procedural time [9,13].

Existing literature on intraoral scanning systems presents a spectrum of findings that reinforces the value of generational progress. An in vitro assessment of trueness and precision in full-arch impressions, for example, noted that later models achieved trueness levels around 23.25 μm, marginally surpassing those of prior iterations at 25.55 μm in preparations for short-span fixed prostheses [7]. This parallels the current study's demonstration of greater agreement in the newer system, suggesting that cumulative enhancements in hardware and software yield measurable gains. Likewise, a comparative evaluation of multiple scanning devices indicated that recent generations, including those with AI integration, maintained high precision without significant inter-device variances and excelled in trueness, particularly in scenarios involving irregular surfaces or angulations [18]. A 2025 study on implant angulation effects found that IOS without auxiliary devices exhibited higher deviations in tilted configurations, but prefabricated aids could achieve clinically acceptable accuracy comparable to photogrammetry [15]. This underscores the potential benefits of newer IOS like the Medit i700 in handling angulated implants, as observed in our results with minimal bias.

Economic aspects are also pertinent: higher-priced advanced devices often include premium sensors that improve light acquisition and noise suppression, as reflected in the lower bias of the newer model [13]. Autonomous functionalities like AI-based artifact mitigation curtail operator-induced inconsistencies [10]. Conversely, earlier models may show greater susceptibility to environmental factors or scan path inconsistencies [19]. In our study, the wider LoA for Dentsply Sirona may indicate such vulnerabilities, particularly in replicating precise distances, which could affect prosthetic fit in single-implant cases [14]. Recent 2025 research comparing conventional impressions and IOS for All-on-Four implants reported that while conventional methods offer better trueness, scanners like TRIOS 5 provide superior precision, although overall deviations may necessitate verification tools for clinical use [16]. Our findings extend this by showing generational differences in single-implant accuracy, where the Medit i700's performance edge (bias reduction from 0.05 mm to 0.02 mm, LoA span from 0.75 mm to 0.24 mm) highlights enhanced trueness and precision without additional aids.

Comparing intraoral scanning systems from different generations is essential to evaluate how technological advancements translate into clinical benefits. This study demonstrated a significant improvement in accuracy with the newer generation scanner, achieving a reduction in bias (0.02 mm versus 0.05 mm) and a narrower range of agreement limits (0.24 mm span versus 0.75 mm) compared to the older model, highlighting enhanced trueness and precision. Newer generation scanners, with superior optical quality, AI-driven artifact correction, and improved handling of undercuts and tilted implants, also offer greater ease of use; for instance, the Medit i700’s lightweight design (245g) and reversible tip enhance maneuverability compared to the bulkier Dentsply Sirona Primescan (~457g), which can feel less comfortable in tight spaces. Additionally, newer systems like the Medit i700 provide faster scanning speeds (up to 70 frames per second (fps), full-arch in 18-40 seconds) versus the Primescan’s slightly slower processing (under 60 seconds), streamlining workflows and improving patient comfort. In this study, six patients with 10 MIS implant sites underwent intraoral and model-based measurements of scan body-to-tooth and intercuspal distances, revealing the newer scanner’s superior accuracy (ICC = 1.00, bias = 0.02 mm) over the older (ICC = 0.999, bias = 0.05 mm), with comparable intercuspal results, thereby underscoring the advantages of generational advancements. In our clinical context, these insights therefore suggest that newer IOS could improve passive fit in prosthetics, reducing biological complications like peri-implantitis from misfit-induced stress [5]. Moreover, an umbrella review from 2025 confirmed IOS reliability for short-span implants but noted challenges in edentulous arches, advocating for careful factor consideration in workflows [9]. Our results, focused on single implants with adjacent teeth, support this for dentate scenarios, where the Medit i700's excellent ICC (1.00) indicates near-perfect agreement with gold standards.

The novelty of this study lies in its focused comparison of intraoral scanners from different generations (2021 Medit i700 vs. 2019 Dentsply Sirona) in a clinical context, using direct intraoral measurements as the gold standard. Unlike many in vitro studies, this cross-sectional clinical evaluation directly assesses devices with varying manufacturing dates and costs for single-implant prosthetics, highlighting how rapid technological advancements (e.g., AI integration, enhanced optics) translate to superior trueness and precision. Moreover, it uniquely evaluates scan body-to-tooth distances, which are critical for prosthesis design but underexplored in prior research. This addresses literature gaps, as most studies overlook clinical variables like soft-tissue mobility while emphasizing full-arch or edentulous models. The use of blinded measurements, ICC, and Bland-Altman analysis in a real-patient setting also adds robustness, contrasting with dominant in vitro approaches. It provides actionable insights into generational differences, potentially guiding future scanner development and clinical guidelines.

Clinical significance

This study has practical implications for implant dentistry by demonstrating that newer-generation intraoral scanners, like the Medit i700, offer superior accuracy in replicating implant positions compared to older models such as the Dentsply Sirona CEREC Omnicam. In clinical practice, this translates to: (1) Improved prosthetic fit and patient outcomes: Accurate digital impressions reduce misalignments (as small as 50-100 μm), minimizing stress on the implant-bone interface and lowering risks of biological (e.g., peri-implantitis) or mechanical failures. This could lead to longer-lasting restorations and fewer revisions, enhancing patient satisfaction and reducing chair time. (2) Workflow efficiency: Clinicians can select scanners with advanced features (e.g., AI-driven artifact removal, faster scanning) to streamline digital workflows, especially for single-implant cases with adjacent teeth. This supports better decision-making in equipment investment, potentially justifying higher costs for premium devices that yield measurable precision gains. (3) Real-world application in daily practice: For periodontists and prosthodontists, the findings endorse preferring newer scanners in precision-demanding scenarios, such as posterior regions or subgingival margins, where variability in older models could compromise prosthesis design. Overall, it promotes evidence-based adoption of digital technologies to optimize implant prosthetics in routine clinical settings.

Limitations

While the study provides valuable insights, it has several limitations. Only 10 implant sites from six patients were evaluated, which may not fully capture population variability or detect subtle differences, limiting statistical power. Moreover, this may not capture broader variability. In vitro measurements performed on 3D-printed models from STL files could introduce possible inaccuracies from printer resolution or material properties. The focus of this study was on single MIS implants in stable, dentate conditions with adjacent natural teeth; these results may therefore not extend to multi-unit restorations, edentulous arches, angulated implants, or other implant systems/brands. Intraoral factors like saliva, tongue movement, or patient motion were not fully accounted for, which could potentially overestimate accuracy in real-world scenarios [6]. Additionally, the study did not evaluate long-term clinical outcomes, such as prosthesis survival, fit over time, or patient-reported outcomes, which could be influenced by these accuracy differences [9].

Future directions

To build on this study, future research should address these limitations and expand the scope of intraoral scanner evaluations in implant dentistry. This could be through: (1) Larger and more diverse cohorts: Conduct multicenter studies with bigger sample sizes, including varied patient demographics, implant types (e.g., bone-level vs. tissue-level), angulations (e.g., 30° tilts), and arch spans (e.g., full-arch or All-on-Four cases) to enhance generalizability. (2) In vivo and longitudinal assessments: Incorporate real-time clinical trials evaluating long-term outcomes like prosthesis fit, survival rates, peri-implant health, and complication rates post-fabrication, using patient follow-ups over months or years. (3) Integration of auxiliary tools and variables: Investigate the impact of adjunctive devices (e.g., prefabricated scan aids) on accuracy in challenging scenarios, and simulate dynamic oral conditions (e.g., saliva, movement) to better mimic real-life use. (4) Software and hardware updates: Examine how firmware/software upgrades affect older scanners' performance, and compare emerging technologies (e.g., next-gen AI-enhanced scanners) against current benchmarks. (5) Comparative and economic analyses: Perform head-to-head trials with additional scanners and include cost-benefit analyses to guide clinical adoption, alongside hybrid workflows combining digital and conventional impressions. (6) Advanced metrics and AI applications: Explore machine learning for automated accuracy prediction, root mean square (RMS) deviations in complex cases, and integration with CAD/computer-aided manufacturing (CAM) systems for end-to-end prosthetic workflows [15,17]. Systematic reviews synthesizing generational advancements could also inform guidelines.

## Conclusions

This study highlights the enhanced accuracy of newer-generation intraoral scanning systems in contrast to their older counterparts in reproducing implant positions for prosthetic fabrication. Demonstrating robust concordance with intraoral standards and showing limited fluctuation, newer intraoral scanning systems position themselves as advantageous in implantology. Their merits, as a result of being produced more contemporarily, as well as their progressive attributes, including superior quality, integrations with artificial intelligence tools, proficient management of undercuts and tilted scan bodies, and generational evolutions, propose avenues for better clinical productivity. Although both generational strata align with clinical practice norms, the accuracy of advanced models may refine digital procedures. Further studies are needed to confirm these benefits in diverse scenarios.
